# Analysis of the efficacy of HIV protease inhibitors against SARS-CoV-2′s main protease

**DOI:** 10.1186/s12985-020-01457-0

**Published:** 2020-11-26

**Authors:** Mohamed Mahdi, János András Mótyán, Zsófia Ilona Szojka, Mária Golda, Márió Miczi, József Tőzsér

**Affiliations:** 1 Laboratory of Retroviral Biochemistry, Department of Biochemistry and Molecular Biology, Faculty of Medicine, University of Debrecen, Egyetem tér 1. Life Science Building, Debrecen, 4032 Hungary; 2 Doctoral School of Molecular Cell and Immune Biology, University of Debrecen, Debrecen, Hungary

**Keywords:** SARS-CoV-2, Protease, HIV protease inhibitors, In vitro assay, Inhibition profiling

## Abstract

**Background:**

The pandemic caused by severe acute respiratory syndrome coronavirus 2 (SARS-CoV-2) has resulted in millions of infections worldwide. While the search for an effective antiviral is still ongoing, experimental therapies based on repurposing of available antivirals is being attempted, of which HIV protease inhibitors (PIs) have gained considerable interest. Inhibition profiling of the PIs directly against the viral protease has never been attempted in vitro*,* and while few studies reported an efficacy of lopinavir and ritonavir in SARS-CoV-2 context, the mechanism of action of the drugs remains to be validated.

**Methods:**

We carried out an in-depth analysis of the efficacy of HIV PIs against the main protease of SARS-CoV-2 (M^pro^) in cell culture and in vitro enzymatic assays, using a methodology that enabled us to focus solely on any potential inhibitory effects of the inhibitors against the viral protease. For cell culture experiments a dark-to-bright GFP reporter substrate system was designed.

**Results:**

Lopinavir, ritonavir, darunavir, saquinavir, and atazanavir were able to inhibit the viral protease in cell culture, albeit in concentrations much higher than their achievable plasma levels, given their current drug formulations. While inhibition by lopinavir was attributed to its cytotoxicity, ritonavir was the most effective of the panel, with IC_50_ of 13.7 µM. None of the inhibitors showed significant inhibition of SARS-CoV-2 M^pro^ in our in vitro enzymatic assays up to 100 µM concentration.

**Conclusion:**

Targeting of SARS-CoV-2 M^pro^ by some of the HIV PIs might be of limited clinical potential, given the high concentration of the drugs required to achieve significant inhibition. Therefore, given their weak inhibition of the viral protease, any potential beneficial effect of the PIs in COVID-19 context might perhaps be attributed to acting on other molecular target(s), rather than SARS-CoV-2 M^pro^.

## Background

In December 2019, a novel severe acute respiratory syndrome coronavirus 2 (SARS-CoV-2) was identified as the etiological agent of viral pneumonia cases that occurred in Wuhan, Hubei Province, China. As of October 29th 2020, the pandemic has resulted in more than 44 million infections, and 1 million deaths worldwide according to the World Health Organization.

There is currently no standardized treatment protocol, and there is no antiviral treatment of proven efficacy recommended for COVID-19. Clinical management of patients is mainly supportive, including supplementary oxygen and mechanical ventilation if needed. However, given the overwhelming burden of the pandemic on national healthcare systems and the global economy, experimental therapies have been attempted, which are predominantly based on the repurposing of FDA approved antivirals, antimalarials, arthritis drugs, and blood plasma derivatives [1].

The HIV protease inhibitors (PIs) lopinavir and ritonavir have gained particular interest, having shown documented in vitro activity against SARS-CoV and the Middle East respiratory syndrome coronavirus (MERS), however, these studies did not identify a molecular target for the drugs, since their efficacy was solely determined based on the inhibition of cytopathic effects or viral replication, respectively [2, 3]. Given the 94.4% identity in amino acid sequence between SARS-CoV and SARS-CoV-2 [4], studying the efficacy of HIV protease inhibitors against SARS-CoV-2 would be of major relevance.

The genome of SARS-CoV-2 encodes for two viral cysteine proteases; nsp3 (papain-like protease) and nsp5 (main protease) [5]. The main protease (M^pro^) of SARS-CoV-2; also named chymotrypsin-like protease (3CLpr), plays a crucial role in the viral life cycle, cleaving the initial polyproteins translated from the viral RNA at at least 11 of its 14 cleavage sites. M^pro^ of SARS-CoV-2 shares 96% sequence identity to that of SARS-CoV. The enzyme consists of three domains; two domains (I and II) which consist of antiparallel β-barrels, and an α-helical domain (I), which is responsible for dimerization and enzymatic activity [6, 7]. Recent structure determination confirmed the similarities between the two enzymes [8].

One potential target for the HIV PIs is the M^pro^. In silico screening identified nelfinavir as its potential inhibitor [9], while lopinavir and ritonavir were found to be potential inhibitors of the viral enzyme by molecular dynamics simulation [10].

It is important to note that the HIV protease is a C2-symmetric homodimeric aspartyl protease, composed of two identical subunits that are 99 amino acids each. The active site is located at the interface between the two monomers, and contains the catalytic Asp-Thr-Gly residues [11]. M^pro^ on the other hand, is a cysteine protease that can also potentially be targeted by peptide mimetics. Given the structural difference between the two proteases, the efficacy of HIV protease inhibitors against SARS-CoV and SARS-CoV-2 is questionable.

Previous studies reported that a combination of lopinavir/ritonavir and ribavirin was effective against SARS-associated coronavirus, with concentrations of 4 µg/ml and 50 µg/ml, respectively [12]. However, a recent clinical trial of 99 patients with laboratory-confirmed SARS-CoV-2 infection who were treated with lopinavir–ritonavir concluded that no significant benefit was observed in the treated group, compared to those who received standard care [13]. Recently, a short communication reported that lopinavir inhibited SARS-CoV-2 replication in Vero E6 cells with IC_50_ of 26.63 μM, ritonavir, however, showed no inhibition of viral replication [14].

Early in vitro reports from China showed that darunavir inhibited SARS-CoV-2 replication, although at a very high concentration (300 µM) [15]. Clinical trials are currently ongoing [16].

Our aim was to test the efficacy of a panel of HIV PIs against SARS-CoV-2 M^pro^, using a cell culture-based model. In this study, we determined the IC_50_ of the PIs with the aid of a dark-to-bright GFP substrate system that had been developed and applied previously for the investigation of caspases [17]. Moreover, in vitro enzymatic inhibitory assays were also carried out using purified M^pro^ and an oligopeptide substrate representing the AVLQ*SGFR cleavage site of SARS-CoV-2 polyprotein 1 ab (PP1ab).

## Materials and methods

### Plasmids and inhibitors

Coding sequence of SARS-CoV-2 M^pro^ (GenBank: MT291835.2) was cloned into pcDNA3.1( +) mammalian expression plasmid using BamHI/EcoRI restriction sites to create the SARS-CoV-2 M^pro^ coding plasmid; thereafter referred to as CoV-2 M^pro^. The coding sequence of a dark-to-bright GFP reporter substrate; thereafter referred to as PR-Sub, was also cloned into pcDNA3.1( +) plasmid. The PR-Sub was designed to contain a sequence representing the N-terminal autoproteolytic cleavage site of SARS-CoV-2 M^pro^ (TSAVLQ*SGFRKM); corresponding to the nsp4/nsp5 cleavage site, between the GFP and the Influenza A/M2 protein hydrophobic tail (CNDSSDPLVVAASIIGILHLILWILDRL). For in vitro expression of the protease, the coding sequence of His_6_-tagged M^pro^ was cloned into pET11a bacterial expression plasmid using NdeI and BamHI enzymes. The above mentioned expression constructs were obtained using the gene synthesis service of GenScript.

The protease inhibitors darunavir, saquinavir, lopinavir, tipranavir, indinavir sulfate, and atazanavir sulfate were obtained through the NIH AIDS Reagent Program, Division of AIDS, NIAID, NIH. Ritonavir was obtained from Abbott laboratories, nelfinavir from Agouron, and atazanavir from Bristol-Myers Squibb.

A synthetic oligopeptide used in our in vitro enzymatic assay representing the N-terminal autoproteolytic cleavage site of SARS-CoV-2 M^pro^ (AVLQ*SGFR) was obtained from a peptide synthesis service (BioBasic).

### Analysis of transfection efficiency and proteolysis

293 T human embryonic kidney cells (HEK-293 T) (Invitrogen) were maintained in T-75 flask in 15 mL Dulbecco’s modified Eagle’s medium (DMEM) (Sigma-Aldrich) supplemented with 10% fetal bovine serum (FBS), 1% glutamine and 1% penicillin–streptomycin. Cells were transfected at 70% confluency with 5 µg of either PR-Sub, or CoV-2 M^pro^ plus PR-Sub plasmids using PEI method [18]. After 24 h incubation, GFP fluorescence was analyzed by flow cytometry using FACS Calibur (BD Biosciences).

### Inhibition profiling in cell culture

On the day of transfection, HEK-293 T cells were split and transferred into a 48-well plates (30,000 cells/well) containing serial dilutions of the inhibitor ranging from 200 µM to 5 nM in a total volume of 200 μL DMEM/well, supplemented with 10% FBS, 1% glutamine and 1% penicillin–streptomycin. After 3 h incubation at 37 °C, cells were transfected with 300 ng of CoV-2 M^pro^ and PR-Sub plasmids using lipofectamine LTX reagent (Thermo Fisher Scientific), then the cells were incubated for 24 h. GFP fluorescence was then measured by flow cytometry using FACS Calibur. The results were analyzed by FlowJo Software Version 10 (Becton, Dickinson and Company; 2019). Calculations of IC_50_ were performed using GraphPad Prism 5.0 (GraphPad Software, Inc).

### Cell viability assay

The day before the assay, HEK-293 T cells were split into a 96-well plates (20,000 cells/well) containing serial dilutions of the inhibitor ranging from 200 µM to 100 nM in a total volume of 200 μL DMEM/well, supplemented with 10% FBS, 1% glutamine and 1% penicillin–streptomycin. The next day, the medium was replaced with 100 μL of OPTI-MEM culture media supplemented with 10% FBS, and 10 µL of the 12 mM 3-(4,5-dimethylthiazol-2-yl)-2,5-diphenyltetrazolium bromide (MTT) stock solution was added to the cells. After 4 h incubation at 37 °C, 85 µL of supernatant was removed, and 50 µL of DMSO was added to the cells followed by incubation for 10 min at 37 °C. Absorbance was measured at 540 nm using Synergy H1 Hybrid Multi-Mode Reader (Agilent).

### Expression and purification of SARS-CoV-2 M^pro^

The heat-shock transformed BL21(DE3) cells containing the pET11a-His_6_-M^pro^ plasmid were incubated in 30 ml Luria–Bertani (LB) medium supplemented with ampicillin (100 µg/ml final concentration) at 37 °C for 16 h. The pre-cultured medium was inoculated into 470 ml LB (100 µg/ml ampicillin) and further incubated at 37 °C. Protein expression was induced by isopropyl β-D-1-thiogalactopyranoside (IPTG) (1 mM final concentration) when the OD_600_ reached 0.6–0.8. After 3 h incubation, cells were pelleted by centrifugation at 4 °C for 20 min at 5,000 × g (Sorvall Lynx 4000, Thermo Fisher Scientific), the cell pellet was resuspended in 10 ml buffer A (20 mM Tris, 150 mM NaCl, 10 mM imidazole, pH 7.5) and lysed by sonication on ice (Branson Sonifier 450). After a repeated centrifugation at 4 °C for 20 min at 10,000 × g, the pellet was discarded and His_6_-M^pro^ was purified from the supernatant by Ni-chelate affinity chromatography with the aid of His-Trap Column (GE Healthcare) using Äkta Prime instrument (Amersham Pharmacia Biotech). The column was equilibrated and washed with buffer A, the His_6_-M^pro^ protein was eluted under 20 column volume with a linear gradient of imidazole (0—500 mM imidazole) using buffer B (20 mM Tris, 150 mM NaCl, 500 mM imidazole, pH 7.5). Afterwards, the purification buffer was exchanged to buffer C (20 mM Tris, 50 mM NaCl, 2 mM CaCl_2_ pH 7.5) using Amicon Ultra centrifugal filters (10 K, Merck Millipore) and then the protein was incubated with Factor Xa (10 µg FXa/ mg protein, BCXA-1060, Haematologic Technologies) at 16 °C for 16 h to remove His_6_ fusion tag. Before the next purification step, the buffer was changed to buffer D (20 mM Tris, 1 mM DTT, pH 8.0) and the protein was further purified by ion-exchange chromatography using HiTrap Q FF column (GE Healthcare) equilibrated with buffer D, and eluted with buffer E (20 mM Tris, 1 M NaCl, 1 mM DTT, pH 8.0) under 20 column volume with a linear gradient. The high-purity fractions of the untagged M^pro^ were dialyzed against buffer F (20 mM HEPES, 120 mM NaCl, 0.4 mM EDTA, 4 mM DTT, 20% glycerol pH 6.5), and stored at -20 °C in a small-volume aliquots.

### In vitro protease assay

The AVLQ*SGFR oligopeptide was dissolved in distilled water and was used as substrate in activity measurements to test the inhibitory potential of the PIs.

The cleavage reactions contained 10 µL reaction buffer (20 mM Tris, 100 mM NaCl, pH 7.8), 4.8 µL oligopeptide substrate (1.37 mM final concentration), and 0.2 µL DMSO (in control samples) or 0.2 µL of the inhibitor (diluted in DMSO). For inhibitor screening, inhibitors were applied in 100 µM final concentration. Reactions were initiated by the addition of 5 µL of M^pro^ in a final total protein concentration of 0.12 µM, and the mixtures were incubated at 37 °C for 10 min. The reactions were terminated by the addition of 180 µL 1% trifluoroacetic acid (TFA). The cleavage products were detected using high performance liquid chromatography (HPLC), utilizing a 0–100% water-acetonitrile gradient in the presence of TFA using Merck Hitachi instrument. Relative activity was determined at less than 20% substrate hydrolysis. Activity measured in the presence of DMSO was considered to be 100%. While no potent inhibitor of M^pro^ was available to perform active-site titration, 100% activity was assumed for the enzyme.

### Modeling

Homology modeling of dark-to-bright GFP substrate was performed using Phyre2 web portal [19]. 97% of residues were modelled at > 90% confidence. Structural figures were prepared PyMol Molecular Graphics System (Version 1.3 Schrödinger, LLC).

## Results

### Inhibition profiling in cell culture

**Fig. 1:**
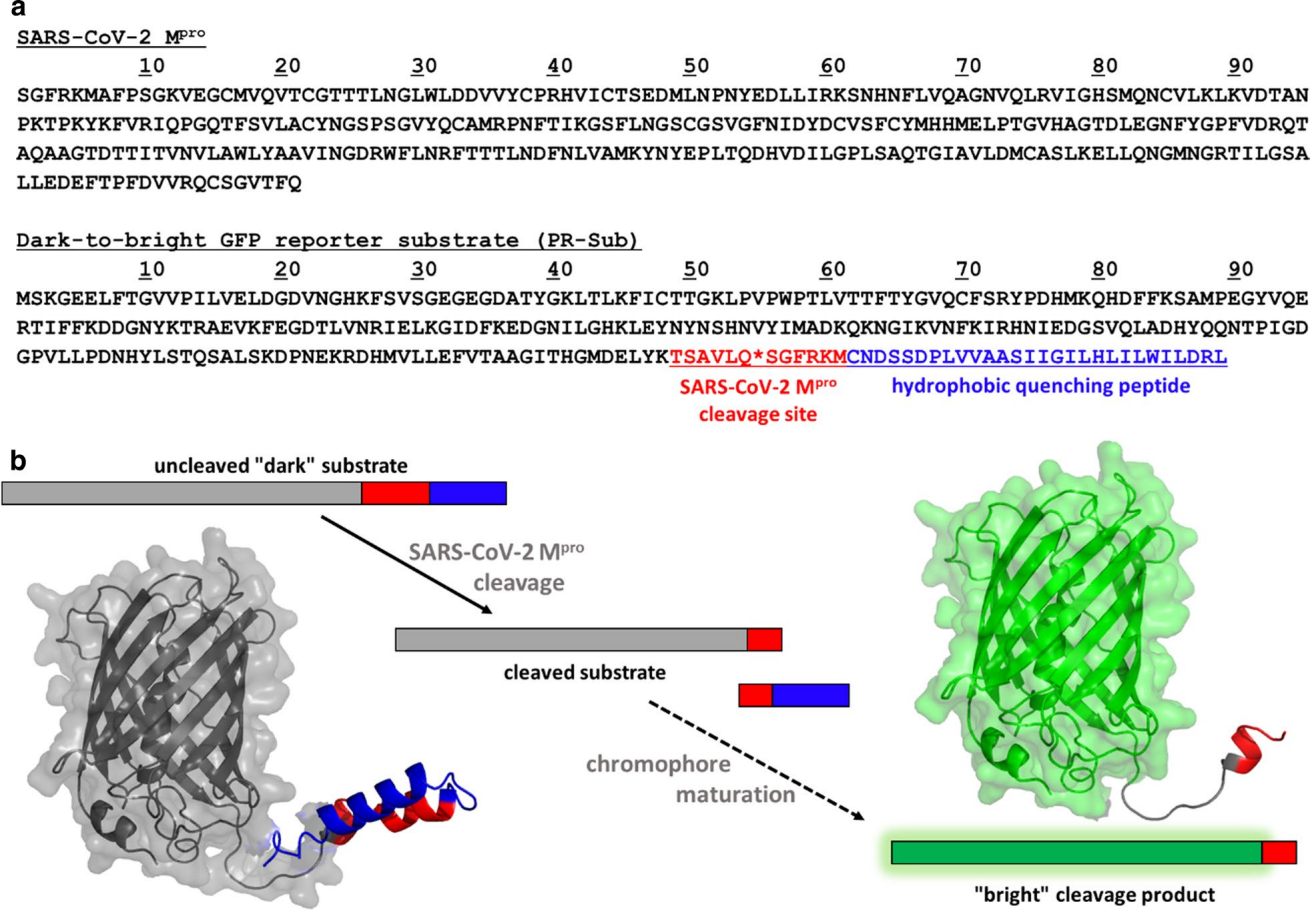
The dark-to-bright reporter system utilized to investigate M^pro^ activity in cell culture. **a** Sequences of M^pro^ and the reporter substrate (PR-Sub). **b** Schematic representation of the dark-to-bright reporter system. Homology model structure of PR-Sub is also shown

To measure M^pro^ activity in cell culture experiments, we applied a modified version of a dark-to-bright GFP reporter substrate [17] which was adapted in this study for SARS-CoV-2 M^pro^. The recombinant substrate consists of an N-terminal GFP, followed by a natural proteolytic cleavage site of SARS-CoV-2 polyprotein, and a C-terminal hydrophobic tail. Proteolysis at the inserted cleavage site releases the tail that serves as a hydrophobic quencher of fluorescence and facilitates tetramerization of GFP, which prevents chromophore maturation; the fluorescence is restored upon proteolysis (Fig. 1).

**Fig. 2:**
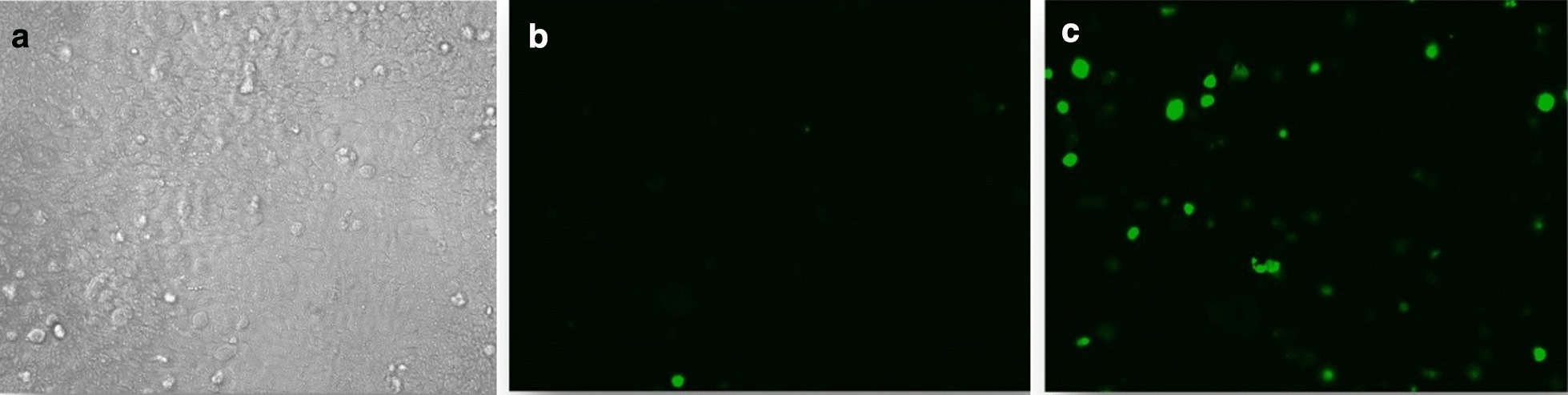
Optimization of HEK-293 T cell transfection with SARS-CoV-2-M^pro^ and the dark-to-bright GFP substrate. **a** Cells transfected with PR-Sub and CoV-2 M^pro^ under native microscopic light. **b** Visualization of cells transfected with PR-Sub under fluorescent microscope. **c** Visualization of cells transfected with PR-Sub and CoV-2 M^pro^ under fluorescent microscope. Co-transfection with both plasmids resulted in 28–34% GFP fluorescence

Firstly, we optimized the transfection of HEK-293 T cells for the use of the SARS-CoV-2 M^pro^ and the dark-to-bright GFP substrate. Transfection of cells with only the PR-Sub plasmid resulted in a maximum of 1% background fluorescence after 24 h incubation. When cells were transfected with both the PR-Sub and CoV-2 M^pro^ plasmids, GFP fluorescence ranged from 28–34%, indicating processing of the substrate and the activity of the protease (Fig. 2).

We then analyzed the inhibition efficacy of a panel of HIV PIs against SARS-CoV-2 M^pro^. While none of the inhibitors was able inhibit the viral protease in nanomolar concentration; which is expected for effective transition state analogs, in micromolar range, ritonavir was the most effective (IC_50_ = 13.7 ± 1.1 µM). Saquinavir, darunavir, and atazanavir were also able to inhibit SARS-CoV-2 M^pro^ at higher concentrations (Table 1).

**Table 1:** Results of inhibition profiling of HIV protease inhibitors against SARS-CoV-2 M^pro^ in cell culture. Data are calculated from triplicate experiments. IC_50_ for lopinavir was indeterminable accurately due to high cytotoxic effects in HEK-293 T cells

Inhibitor	IC_50_ (µM)	Standard error
Lopinavir + Ritonavir	10.9	± 1.1
Ritonavir	13.7	± 1.1
Saquinavir	31.4	± 1.2
Darunavir	36.1	± 1.2
Atazanavir	60.7	± 2.5
Lopinavir	Indeterminable	
Indinavir	No inhibition (up to 200 µM)	
Nelfinavir	No inhibition (up to 200 µM)	
Tipranavir	No inhibition (up to 200 µM)	

**Fig. 3:**
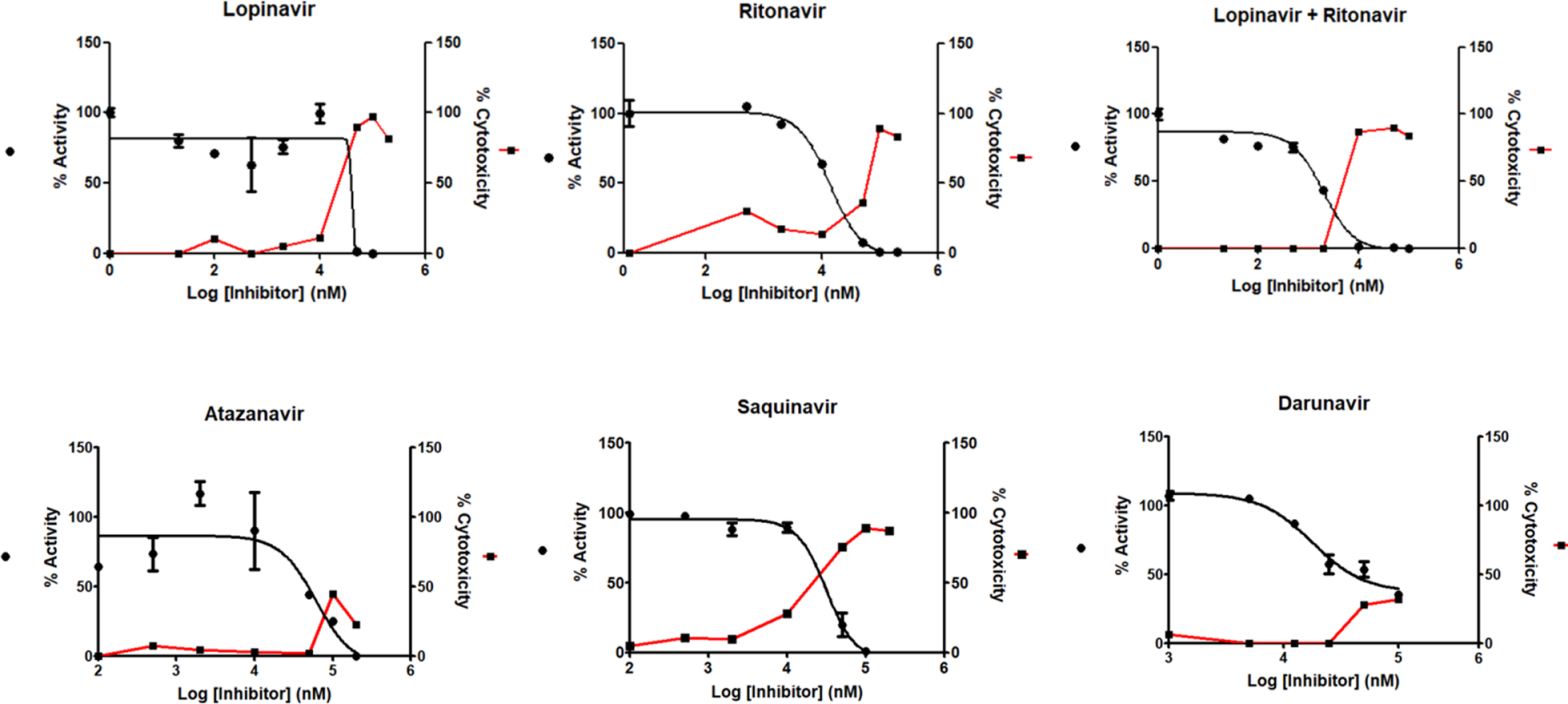
Determination of IC_50_ in cell culture. Relative activity (%) is plotted on the left Y axis versus logarithmic transformation of the inhibitor's concentration (nM). Percentage of cytotoxicity is plotted on the right Y axis. Error bars represent SD (n = 3)

Although a combination of lopinavir and ritonavir resulted in better inhibition of the viral enzyme as compared to ritonavir alone (10.9 µM vs. 13.7 µM, respectively), when we carried out cell viability assays after treatment of the cells with the inhibitors, interestingly, inhibition by lopinavir was found to be a result of the high cytotoxicity observed at concentrations above 50 µM (90%) (Fig. 3).

In the case of ritonavir and saquinavir, cytotoxicity of > 50% was only observed at concentrations above 50 µM. High concentrations of darunavir and atazanavir on the other hand, were well tolerated by HEK-293 T cells.

Indinavir, nelfinavir and tipranavir however, failed to inhibit SARS-CoV-2 M^pro^, even at 200 micromolar concentration of the inhibitors.

### In vitro enzymatic assay

**Fig. 4:**
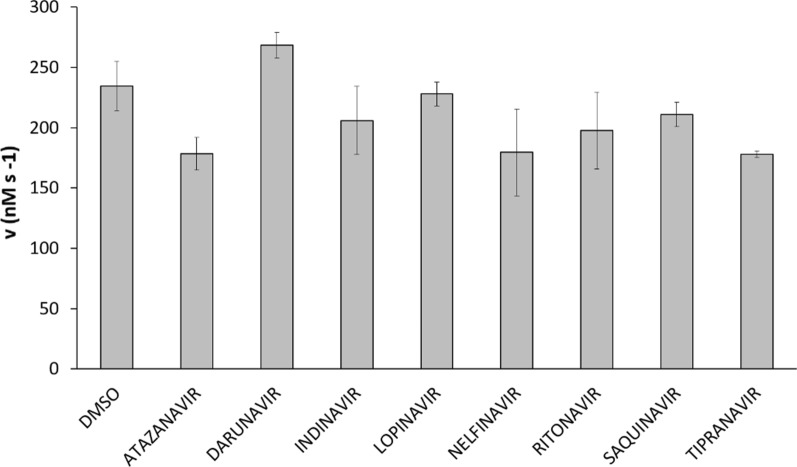
Inhibition profiling using an enzymatic assay. Results show that none of the inhibitors showed significant inhibition of SARS-CoV-2 M^pro^ at a concentration of 100 µM in the reaction. The control reaction contained DMSO without a protease inhibitor. Error bars represent SD (n = 2)

Following expression and purification of the untagged M^pro^, we determined its catalytic activity after incubation with the AVLQ*SGFR oligopeptide substrate. Cleavage position within the substrate was determined using high-performance liquid chromatography coupled to electrospray ionization time-of-flight mass spectrometry (HPLC-ESI-TOF MS) (Additional file 1: Fig. 1). Inhibition profiling of the PIs was carried out after incubation of the inhibitors along with M^pro^ and the substrate, and the relative efficacies of PIs were compared. None of the inhibitors showed a significant inhibition of the M^pro^ in vitro (*p* values > 0.05) (Fig. 4).

## Discussion

To our knowledge, direct determination of the inhibition efficacy of HIV PIs against SARS-CoV-2 M^pro^ in cell culture has not yet been published, although many in silico studies analyzing interaction between SARS-CoV-2 M^pro^ and potential inhibitors were published [20–26] (Additional file 1: Table 1). Antiviral assays using lopinavir and ritonavir in Calu-3 cells were previously carried out for MERS-CoV, and the IC_50_ for lopinavir, ritonavir and their combination was 11.6, 24.9, and 8.5 µM respectively [3]. In our analysis, we found that a combination of lopinavir plus ritonavir achieved the lowest IC_50_, this however was due to the high cytotoxicity of lopinavir, and not as a result of direct inhibition of SARS-CoV-2 M^pro^. Ritonavir on the other hand was much more tolerable than lopinavir, and achieved the lowest IC_50_. This should be taken into consideration, given the current drug formulation of lopinavir which is in combination with ritonavir, where ritonavir is used as a pharmacokinetic enhancer due to its inhibition of the cytochrome P450 3A4 isoenzyme, thereby increasing the bioavailability of lopinavir [27]. As a result, administration of ritonavir in combination with other PIs was found to decrease its minimum blood plasma concentration level, as compared to the generic formulation of the drug [28]. Also, while our result regarding ritonavir was in direct contrast to what Choy et al*.* reported in their short communication [14], we believe that difference in methodologies is to blame for this discrepancy, as we examined the efficacy of ritonavir against the viral M^pro^ protease per se.

It is important to note that the IC_50_ of the inhibitors were in the micromolar range which is not considered optimal for the inhibition of the viral enzyme. Previous studies have reported that the minimum concentrations (C_min_) for lopinavir, darunavir, saquinavir, and atazanavir in patient’s serum under antiretroviral treatment was found to be 9.3, 3.3, 3.8, and < 1 µM, respectively [29–32]. It would indeed be challenging to achieve such high plasma levels of the inhibitors in order to block the viral replication, moreover, the cytotoxic effects of some of the inhibitors, in addition to the side effects commonly observed with PIs questions the use of anti-HIV PIs in the context of SARS-CoV-2.

Additionally, using an in vitro enzymatic assay, we were able to directly analyze any potential inhibition of M^pro^ by the HIV PIs. Our results show that none of the inhibitors was able to significantly inhibit M^pro^ in vitro.

A drawback of this study is that we were not able to assess the interaction between the PIs and the papain-like protease of SARS-CoV-2, as our methodology only enabled us to study the viral main protease. Whether or not these exert any inhibitory effect on the papain-like protease is a subject for future studies, although, a similar methodology may be adapted for SARS-CoV-2 papain-like protease, and other proteases as well. Also, while our cell culture assays were not performed in SARS-CoV-2 target cells, our methodology enabled us to directly examine any potential inhibition of the viral M^pro^ by the PIs, therefore, it is unlikely that different results will be obtained in target cells.

## Conclusion

In conclusion, to our knowledge, thorough analysis of the efficacies of PIs against SARS-CoV-2 remains scarce, and the targets of the drugs are yet to be verified. While few studies examined the efficacy of some PIs against the replication of SARS-CoV-2, we set out to study whether or not the inhibitors exert a direct effect on the viral protease. In our experiments, even though some of the PIs developed for the treatment of HIV were able to inhibit SARS-CoV-2 M^pro^, they were only able to do so at high concentrations. The combination of lopinavir plus ritonavir resulted in the lowest IC_50_ in cell culture, albeit at the cost of cellular viability. Although, darunavir and atazanavir required a much higher concentration to achieve the inhibition, cytotoxicity was not observed even at a concentration of 200 µM. It should be noted that there might be other molecular targets for the HIV PIs, as nelfinavir was recently shown to inhibit spike protein-mediated fusion of SARS-CoV-2 [33].

Taking everything into consideration, the use of HIV PIs in the context of COVID-19 might be of limited clinical potential, beneficial effects of which might perhaps be attributed to acting on other molecular target(s), rather than M^pro^ itself. Data from clinical trials will indeed shed more light on their clinical efficacy.

## Supplementary information

**Additional file 1**. Supplementary figure and table.

## Data Availability

All data generated during this study are included in this article and its supplementary information files.

## Abbreviations

SARS-CoV-2: Severe acute respiratory syndrome coronavirus 2
HIV: Human immunodeficiency virus
PI: Protease inhibitor
M^pro^: Main protease
IC_50_: Half maximal inhibitory concentration
COVID-19: Coronavirus disease-2019
WHO: World Health Organization
FDA: Food and Drug Administration
MERS: Middle East respiratory syndrome
PR: Protease
PEI: Polyethylenimine
GFP: Green fluorescent protein
FACS: Fluorescence-activated cell sorting
HEK-293 T: Human embryonic kidney 293 cells
DMSO: Dimethyl sulfoxide
MTT: 3-(4,5-Dimethylthiazol-2-yl)-2,5-diphenyltetrazolium bromide
IPTG: Isopropyl β-D-1-thiogalactopyranoside
TFA: Trifluoroacetic acid
HPLC-ESI-TOF MS: High-performance liquid chromatography coupled to electrospray ionization time-of-flight mass spectrometry
