# Supplementary material for: Analysis of the efficacy of HIV protease inhibitors against SARS-CoV-2′s main protease
Source: Virol J. 2020 Nov 26;17:190. doi: 10.1186/s12985-020-01457-0 (PMC7689640; doi:10.1186/s12985-020-01457-0)
Supplement: Supplementary file 1 — Additional file 1. Supplementary figure and table. [file 12985_2020_1457_MOESM1_ESM.pdf]

Supplementary figure 1. Determination of susbtrate cleavage and position of the cleavage products by SARS-CoV-2 M^{pro}. His$_6$-tagged purified M^{pro} was incubated with the substrate representing the AVLQ*SGFR cleavage site of SARS-CoV-2 PP1ab. High-performance liquid chromatography coupled to electrospray ionization time-of-flight mass spectrometry (HPLC-ESI-TOF MS) was then used to identify the cleavage products.

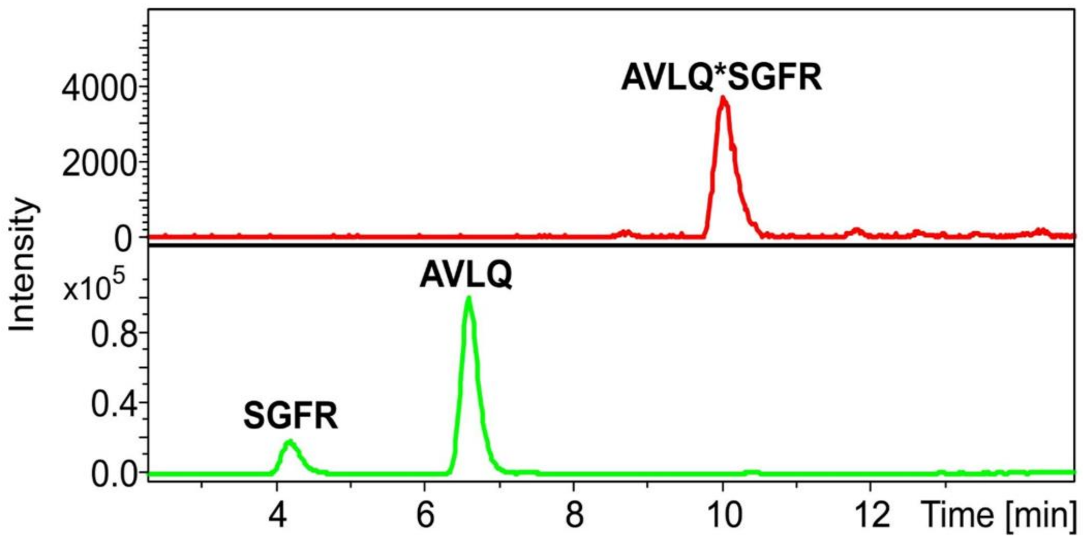

Supplementary table 1. Data compiled from the literature regarding the *in silico* screening of inhibitors against SARS-CoV-2 M^{pro}. Based on the scoring used in the given studies, efficacies of the tested HIV PIs were determined. The inhibitors predicted to be most effective out of the tested ones are shown in grey background. The values determined in the given studies are not fully comparable, as different methods were applied for the calculations. “-“ indicates that the given inhibitor was not included in the study or the obtained value was not reported.

**Reference**	**Value**	**Amprenavir**	**Atazanavir**	**Darunavir**	**Fosamprenavir**	**Indinavir**	**Lopinavir**	**Nelfinavir**	**Ritonavir**	**Saquinavir**	**Tipranavir**
**Ortega et al., 2020**	Binding energy (kcal/mol)	-7.6	-7.2	-8.2	-	-	-9.1	-	-6.9	-9.6	-8.7
**Sang et al., 2020**	Binding energy (kJ/mol)	-	-	-10.24	-	-10.02	-5.49	-	-2.34	-8.26	-5.8
**Calligari et al., 2020**	Vina scoring (kcal/mol)	-7.7	-8.0	-7.6	-7.2	-8.7	-8.1	-7.9	-8.1	-9.3	-8.6
**Pant et al., 2020**	Docking score	-	-	-7.21	-	-7.35	-8.36	-7.44	-8.30	-8.64	-
**Shah et al., 2020**	dock score (5R81)	-6.583	>-6.5	>-6.5	>-6.5	-6.834	-8.44	>-6.5	-6.764	-7.632	>-6.5
**Beck et al., 2020**	predicted K_d (nM)	-	94.94	-	-	-	-	-	204.05	-	-
**Fisher et al., 2020**	binding energy (kcal/mol)	−66.5	-	-	-	-	-	−80.6	-	−71.5	-
